# Diastolic dysfunction and sex-specific progression to HFpEF: current gaps in knowledge and future directions

**DOI:** 10.1186/s12916-022-02650-4

**Published:** 2022-12-27

**Authors:** A. M. L. N. van Ommen, E. Dal Canto, Maarten J. Cramer, F. H. Rutten, N. C. Onland-Moret, H. M. den Ruijter

**Affiliations:** 1 Laboratory of Experimental Cardiology, University Medical Center Utrecht, Utrecht University, 3508 GA Utrecht, The Netherlands; 2 Clinical Cardiology Department, University Medical Center Utrecht, Utrecht University, 3508 GA Utrecht, The Netherlands; 3 Department of General Practice, Julius Center for Health Sciences and Primary Care, University Medical Center Utrecht, Utrecht University, 3508 GA Utrecht, The Netherlands; 4 Department of Epidemiology, Julius Center for Health Sciences and Primary Care, University Medical Center Utrecht, Utrecht University, 3508 GA Utrecht, The Netherlands

**Keywords:** Sex-specific research, Left ventricular diastolic dysfunction, Heart failure with preserved ejection fraction, Pre-clinical disease, Progression, Risk factors

## Abstract

Diastolic dysfunction of the left ventricle (LVDD) is equally common in elderly women and men. LVDD is a condition that can remain latent for a long time but is also held responsible for elevated left ventricular filling pressures and high pulmonary pressures that may result in (exercise-induced) shortness of breath. This symptom is the hallmark of heart failure with preserved ejection fraction (HFpEF) which is predominantly found in women as compared to men within the HF spectrum. Given the mechanistic role of LVDD in the development of HFpEF, we review risk factors and mechanisms that may be responsible for this sex-specific progression of LVDD towards HFpEF from an epidemiological point-of-view and propose future research directions.

## Background

**Table:** 

**Sex and gender**
Although the words gender and sex are often used interchangeably, they have different meanings. Sex refers to biological differences between males and females, for example in reproductive organs and sex hormones, which result in a different physiology and anatomy of the body. Gender refers to a social construct of how men and women, and other gender identities, behave within a certain social or cultural context that relates much to expectations and norms in behavior and attitudes [1]. Both sex and gender are important in clinical research and patient care, however, through different mechanisms [2]. In this review, we will focus on sex and do not specifically discuss the role of gender, although we acknowledge that the two are intimately connected and sex cannot be regarded without recognizing gender.

### Diastolic dysfunction of the heart

**Fig. 1:**
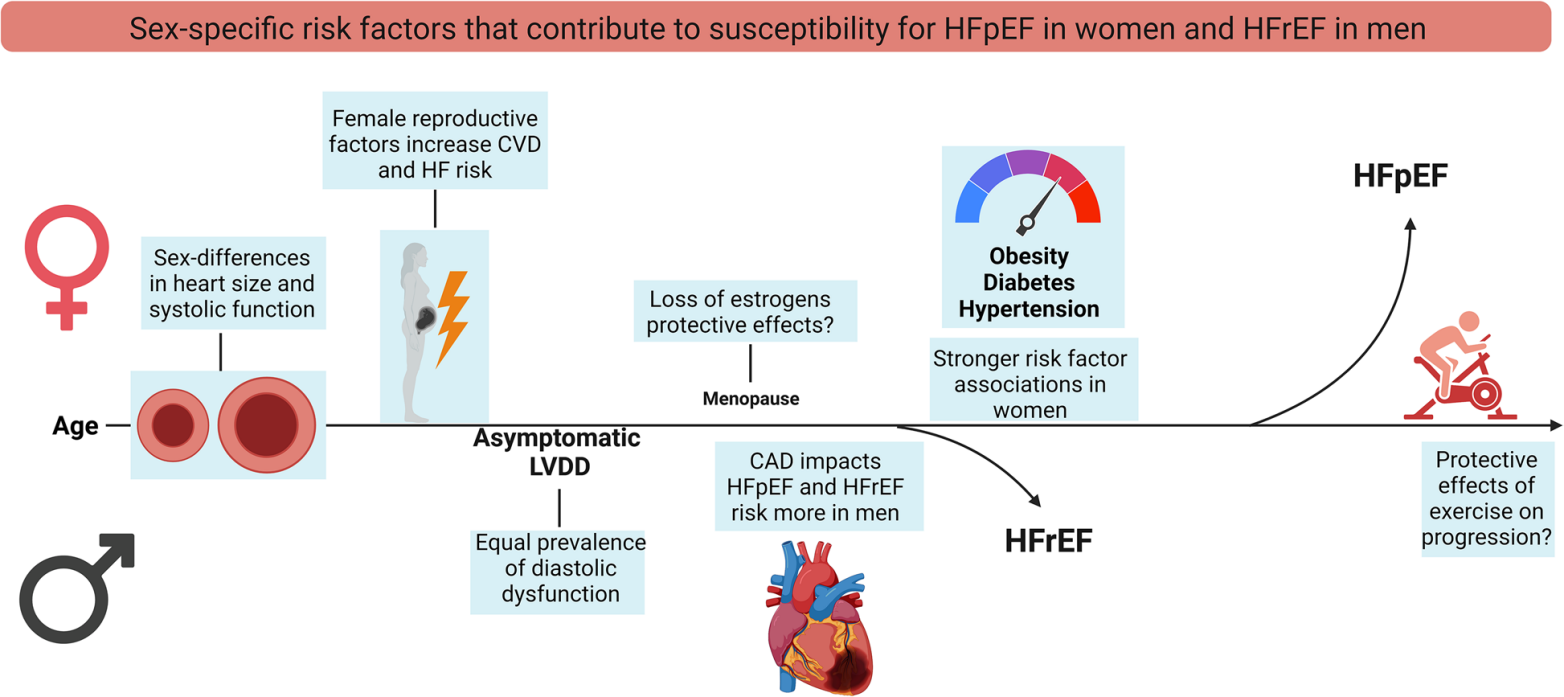
Central illustration. The biological and environmental factors that associate with the development and progression of LVDD and HFpEF in women and men. In women, a smaller heart size results in higher left ventricular ejection fraction and higher global longitudinal strain [16, 17]. Aging is associated with deterioration of diastolic function in both women and men; hence, female reproductive factors may accelerate diastolic function deterioration [18], but further studies are needed on this topic. It is likely that the loss of estrogens due to the menopausal transition contributes to the progression of HFpEF, but targeted therapeutic options in (post-) menopausal women are not yet available. Traditional cardiovascular risk factors also predispose to HFpEF, and obesity, diabetes, and hypertension are examples of risk factors that are more important in women [19–24]. On the other hand, CAD, and the ischemic consequences of CAD, have a larger impact in men with respect to both HFpEF, and HFrEF [25]. Taken together, these biological and environmental factors are likely to explain the susceptibility for HFpEF in women and HFrEF in men but are, inevitably, incomplete

The term left ventricular diastolic dysfunction (LVDD) refers to functional and mechanical problems during diastole, ultimately leading to inadequate filling of the left ventricle. LVDD is caused by a broad range of abnormalities such as altered myocardial relaxation, myocardial stiffness, and left atrial dysfunction [3]. LVDD is an imaging-based finding and does not necessarily cause symptoms. However, LVDD resulting in elevated left ventricular filling pressure, left atrial pressure, and increased pulmonary wedge pressure can cause exercise-induced shortness of breath and reduced exercise tolerance [4]. By the time these symptoms occur, HF is a common diagnosis in both women and men. Prevalence of LVDD ranges between 3.1 and 35% in the general community, these differences being highly dependent on age, and risk factors of the study population, and notably on the different definitions used [5–9]. Multiple studies have shown that there are no important sex-differences in the prevalence of LVDD in community-based cohort studies [5–7] (see Fig. 1). Nevertheless, these studies often fail to report the prevalence of LVDD by sex or by gender. LVDD by echocardiography is evaluated with similar cut-off values for women and men (see Table 1) [3], although for instance some differences in for example E/e′ ratio between women and men were found in healthy populations [10, 11]. Also, guidelines have changed their definition of LVDD over the years, but cut-offs do not differ between women and men. When applying the most recent 2016 guidelines [3] to a French population cohort, the prevalence of LVDD diastolic dysfunction was 0.2% in young individuals of 20 to 40 years of age compared to 1.1% and 3.1% in the age groups 40 to 60 and over 60 years of age [9]. Again, these prevalence numbers were not reported by sex. In addition, the prevalence was much lower compared to earlier guidelines. For example, the prevalence of LVDD was 12.9% in people over 60 years of age when applying the 2009 guidelines [12–15].

To determine diastolic function, imaging is used, and the routine echocardiography report includes information on diastolic function of the heart classifying it as normal, indeterminate, or abnormal using four key parameters listed in Table 1 [3]. For each of these parameters, no sex-specific cut-offs exist and differences between sexes are reported to be small [10, 11, 26, 27]. Diastolic function parameters and all degrees of LVDD were associated with mortality in a large database of 436,360 women and men. Importantly, none of the reported diastolic function measures had a sex-specific association with all-cause mortality. Yet, all-cause mortality is the hardest of all clinical endpoints, and does not reflect sex differences in morbidity such as HFpEF [28]. Symptoms were not taken into account in this study, so it may be that diastolic function parameters have different prognostic consequences to clinically relevant endpoints in women and men.

Alternative echocardiographic parameters can be used to classify LVDD [29, 30]. Some of which differ by sex, e.g., left ventricular global longitudinal strain shows higher normal values in women compared to men [17, 31], and left ventricular mass index (LVMI) has a higher cut-off value for left ventricular hypertrophy in men compared to women (115 g/m^2^ vs 95 g/m^2^) [16]. This reflects inherent sex-differences in cardiac structure and function (see Fig. 1). Men have higher left ventricular mass as compared to women. The difference in LV mass is attributed to the smaller hearts of women, even when indexed to body size, resulting in smaller left ventricular volumes and lower LV mass [32, 33]. To compensate for smaller cavity size, women have a slightly higher left ventricular ejection fraction [16] and higher global longitudinal strain [17, 31]. Still, smaller cavity of the left ventricle is associated with lower cardiac output after indexation to body surface area in healthy women at peak exercise, when compared to healthy men [34]. Furthermore, there is a greater and steeper increase in LV mass with aging women as compared to men [35]. Additionally, there is less cardiomyocyte loss in women during a lifespan [36], and it has been proposed that women are less susceptible to decreases in contractility when afterload increases, as compared to men [37]. Potentially, these dimorphisms in size and function of the heart form the female-specific substrate for a greater susceptibility to further concentric LV remodeling and evolving HFpEF.

**Table 1:** Recommended echocardiography parameters to classify diastolic function in individuals with normal LV ejection fraction according to Nagueh et al. 2016 and 2009 guidelines and known sex differences in these parameters

**Parameter**	**Cut-off 2016** [3]	**Cut-off 2009** [15]^*^	**Sex -differences**
Average E/e′ ratio	> 14	-	± 1 point higher in women [11]
Septal or lateral e′ velocity	< 7 cm/s or < 10 cm/s	< 8 cm/s or < 10 cm/s	no significant differences
TR velocity	> 280 cm/s	-	no significant differences
LAVI	> 34 mL/m^2^	> 34 mL/m^2^	± 2 mL/m^2^ point higher in men [26]
** < 50% positive: normal diastolic function** **50% positive: indeterminate diastolic function** ** > 50% positive: diastolic dysfunction**			

*Abbreviations*: *E/e′ ratio*, the ratio of early mitral valve inflow (E) velocity divided by average e′; *e′*, mitral valve annular early filling tissue Doppler velocity; *TR*, tricuspid regurgitation; *LAVI*, left atrial volume index

^*^After initial assessment of diastolic function using the parameters in the table, it was recommended to take into account E/A ratio (also during Valsalva manoeuver), E wave deceleration time, average E/e′ ratio, and the time difference between reversed pulmonary venous flow (Ar) and A wave duration for detailed LVDD assessment

### Heart failure with preserved ejection fraction (HFpEF)

HFpEF refers to a syndrome in which elevated left ventricular filling pressures and pulmonary pressures resulting from LVDD, cause symptoms and/or signs suggestive of HF, while left ventricular ejection fraction is preserved (≥ 50%) [38]. This might cause an increase in natriuretic peptide levels. The most reported symptom in both women and men with HFpEF is exercise-induced shortness of breath [39]. Heart failure with reduced ejection (HFrEF) fraction is considered the counterpart of HFpEF, since left ventricular ejection fraction is decreased. But, also in HFrEF, LVDD contributes to signs and symptoms through increased left ventricular filling pressures. However, in this review, we will mainly focus on HFpEF.

The diagnosis of HFpEF is complex, also because of the multiple cardiac and non-cardiac comorbidities associated with the disease, such as atrial fibrillation, diabetes, chronic obstructive pulmonary disease (COPD), and renal dysfunction. These comorbidities may be a cause, contributing factor for developing HFpEF, or an alternative diagnosis (“mimic”) for patients presenting with shortness of breath or reduced exercise tolerance. Most of HFpEF comorbidities are hypothesized to contribute to a systemic pro-inflammatory state [40] that can lead to endothelial dysfunction and impaired coronary flow reserve and coronary microvascular dysfunction. The latter were proven to have greater impact on the incidence of major outcomes in women compared to men referred for coronary angiography [41].

### Misdiagnosis and underdiagnosis of HFpEF

We know that aging women from the general population report more exercise-induced complaints, e.g., more severe breathlessness, compared to men [42]. HF is often underdiagnosed in primary care possibly due to limited diagnostic tools such as electrocardiography and measurement of natriuretic peptide plasma levels. On the other hand, spirometry is more readily available upon presentation with shortness of breath. Pulmonary fluid overload may cause pulmonary obstruction and makes it easy to misclassify HF as chronic obstructive pulmonary disease. Indeed, 20% of primary care patients labeled with COPD were diagnosed with HF after undergoing an extensive pulmonary and cardiac assessment, and about half of these HF patients are classified as HFpEF [43]. In men and women aged 65 years or older, who visited their general practitioner for reasons of exertional shortness of breath, resting echocardiography diagnosed 16.5% of men and 15% of women with HF [44]. Interestingly, 76% of these newly detected HF cases were HFpEF cases. Undetected HF was even more prevalent in individuals with diabetes with a prevalence of 27.7%, and again most had HFpEF (83%), with female sex being a predictor of HF [21]. Screening studies like this are scarce and show that HFpEF is frequently underdiagnosed in the elderly. Without a doubt, underdiagnosis or a hampering diagnosis results in lower quality of life and increased health care consumption [45]. Underdiagnosis seems to affect women more often than men, also for myocardial infarction: 30% of electrocardiogram detected myocardial infarction remained unrecognized in women, compared to 16% in men [46]. The more chronic nature of cardiovascular disease in women may go hand in hand with a higher burden of symptoms, or symptoms that are perceived as being atypical or non-cardiac, as shown by a recent meta-analysis of studies in women and men with coronary syndromes [47]. Whether disease presentation is exactly the same in women and men with HFpEF is still unclear.

#### The role of exercise testing in HFpEF diagnosis

In some circumstances, LVDD and HFpEF may only become evident during exercise. In this case, exercise-echocardiography or exercise right heart catheterization are needed for accurate diagnosis [29, 38, 48, 49], since more than half of HFpEF patients with exercise-induced symptoms have normal resting diastolic function [50]. During exercise, women with HFpEF have poorer right ventricular and LV systolic reserve, worse diastolic reserve, lower ventricular vascular coupling, higher systemic and pulmonary vascular resistance, and lower exercise peripheral O_2_ extraction compared to men with HFpEF [51–53]. Finally, while LV ejection fraction is higher in women with HFpEF at rest, during exercise, the rise in stroke volume is blunted, most likely reflecting a greater cardiac afterload [51]. Thus, women with HFpEF appear to, on average, display greater cardiac and systemic impairments than men. It remains unclear, however, whether and to what extent this greater cardiac and systemic impairment in women affects prognosis and drug responsiveness, and whether sex-specific exercise cut-offs are needed for an accurate HFpEF diagnosis. The currently used diagnostic tools for HFpEF all advise additional exercise testing combined with echocardiography or right heart catheterization when diastolic function findings at rest are not conclusive [29, 38, 49].

#### The role of plasma biomarkers in HFpEF diagnosis

Current diagnostic plasma biomarkers for HFpEF are not always useful since natriuretic peptides are often not elevated in HFpEF. In both the general population and in HFpEF studies, women have higher levels of natriuretic peptides than men [54, 55]. Despite these sex differences, current guidelines do not recommend sex-specific cut-offs. Natriuretic peptides levels that fall in the normal range have limited negative predictive value for HFpEF diagnosis [56]. The “natriuretic peptide deficiency” theory hypothesizes that natriuretic peptide levels are low in HFpEF patients due to the inverse relation of natriuretic peptide levels with obesity and high body fat [57, 58]. These are common conditions in HFpEF patients, and both increased breakdown of natriuretic peptides [59], and altered adiponectin signaling [60] may explain low natriuretic peptide levels. Interestingly, subcutaneous adiposity was also correlated with low natriuretic peptides in women, but not in men [61]. Up to now, natriuretic peptides are most commonly used for HFpEF diagnosis. However, proteomics studies are emerging in the HFpEF field [62], and some studies identified sex-specific proteomic signatures [63]. This type of research may help to better understand underlying mechanisms, and to identify (sex-specific) therapeutic targets and more sensitive diagnostic biomarkers [64].

The diagnosis of HFpEF is difficult and often requires (invasive) exercise testing. This makes underdiagnosis of HFpEF common in primary care. Reducing the underdiagnosis of HFpEF will become even more important now that disease-modifying therapies have become available, such as sodium-glucose cotransporter-2 inhibitors [65].

### Epidemiology of heart failure in women and men

The prevalence of established HF worldwide is around 1 to 2.5%, depending on the diagnostic criteria used, and this percentage is equal for women and men [66]. In Western populations, the lifelong risk of HF at the age of 40 years is 21% for women and 20% for men [67] and, at the age of 55 years, 29% and 31% for women and men, respectively [68].

In the period 2000–2010, the incidence of HF in the USA decreased by ~ 5% per year [69], most likely due to better treatment of myocardial infarction [66]. The incidence of HFpEF also decreased, with similar overall rate changes for women and men over 10 years (− 27%; − 2.7% per year), probably due to better treatment of comorbidities [69]. However, mortality and hospitalization rates in HF patients did not decrease over time and remain high [69, 70]. Mortality rates in HF patients are 20% in the first year and reach 50% over 5 years [69]. While total HF prevalence is similar in men and women, women outnumber men with respect to men HFpEF. In community-based studies, women with HF had HFpEF in 67% of the cases, compared to 42% of men with HF having HFpEF [71]. Women account for 55–66% of all HFpEF hospitalizations and only 29–42% of all HFrEF hospitalizations [55, 72, 73]. The proportion of HFpEF cases with respect to overall HF hospitalization is also increasing. In 2010, 39% of hospitalized HF patients had HFpEF, whereas this was 33% in 2005. Unfortunately, this was not reported for women and men separately [74].

The high proportion of women with HFpEF could be accounted for by higher life expectancy in women. Also, a higher prevalence of comorbidities such as chronic kidney disease, hypertension, and valve and lung disease in women with HFpEF explains the female predominance (see Fig. 1) [55]. A study combining data from 4 large population-based cohort studies concluded that women and men have an equal risk to develop HFpEF, after correction for comorbidities and age [75], but that the risk to develop HFrEF is lower in women, as compared to men. Hence, female sex was not an independent risk factor for HF (HR = 0.86 (95% CI: 0.71, 1.04) [76] or HFpEF [25], while male sex was an independent risk factor for HFrEF (HR = 1.84 (95% CI: 1.55, 2.19). However, in a community study among people aged 60 years or over with type 2 diabetes, female sex was an important predictor of previously undetected HF (> 80% HFpEF), but more importantly, in this group of people with type 2 diabetes, the age-stratified prevalence of HFpEF among women was significantly higher than in men [21]. Altogether, despite the finding that sex or gender may not be an independent risk factor for HF development, there is a higher prevalence of HFpEF in women. Therefore, it is important to better understand the role of risk factors contributing to the progression from LVDD to HFpEF that may be associated to female sex.

## Main

### Lack of knowledge on sex-specific risk factors for the progression of diastolic dysfunction towards HFpEF

While the mechanistic role of LVDD in the development of HFpEF is evident, longitudinal data on how LVDD deteriorates towards HFpEF is relatively sparse [77]. As HFpEF is difficult to treat and carries a poor prognosis, preventing HFpEF and limiting disease progression are critical. Therefore, predicting progression from LVDD to HFpEF is key to guide aggressive risk factor management and earlier intervention. Eleven longitudinal studies described the progression of LVDD towards HF (Table 2). The percentage of women participating ranged from 19 to 61%. The proportion of participants with mild to severe diastolic dysfunction that developed HF ranged from 0.8 to 37% during a follow-up time of 1.2 to 11 years. Out of these 11 studies, only one distinguished between HFpEF and HFrEF when investigating the progression of LVDD towards HF [8]. In this study, with a median follow-up of 11 years, LVDD was present in 36% of the participants at baseline. These participants had a high risk of developing HFpEF (HR = 1.88, 95% CI: 1.13, 3.13) even after correction for age, sex, body mass index, systolic blood pressure, hypertension treatment, cholesterol levels, diabetes mellitus, prior myocardial infarction, and valvular heart disease [8]. The main risk factor for progression in this study was airflow limitation which could be a manifestation of sub-clinical pulmonary disease, leading to low-grade inflammation [8]. Further risk factors for the progression of LVDD or pre-clinical HF to overt HF were older age [78–80], hypertension [78, 81], peripheral vascular disease [81], diabetes [78], coronary artery disease [78], (subclinical) renal impairment [8, 79], anemia [8], and the Charlson comorbidity score [80]. These risk factors are exemplary for the multi-organ involvement of the HFpEF syndrome. Given the higher prevalence of HFpEF in women, it may be that this comorbidity-driven progression of LVDD towards HFpEF is sex-specific (see Fig. 1). On the other hand, the observation that female sex was not unequivocally an independent risk factor for HF(pEF) questions this idea. This is indeed also confirmed by three studies that reported that sex was not significantly influencing the progression from LVDD towards HF [80, 82, 83], suggesting that the risk of progression from LVDD to HFpEF is similar in women and men. Nevertheless, most studies do not test for effect modification by sex, do not perform sex-stratified analyses, or study female-specific associations, as was previously also shown in a systematic review on LVDD/HFpEF [84]. This is important because stronger associations of comorbid conditions for one of the sexes may lead to an absent relation of sex itself in multivariable analyses correcting for comorbidities. We therefore highlight several areas in HFpEF research in which the incorporation of sex and gender analyses are likely to enable advancements in the field.

**Table 2:** Studies investigating the progression from LVDD towards overt heart failure

First author, year, cohort name (reference number)	Population under investigation	Number of individuals (% women)	Mean age in years	Follow-up in years	Percentage of individuals that developed heart failure (stage C/D)	Determinants of progression towards heart failure	Distinction between HFpEF and HFrEF	Sex-stratified analyses	Sex included in multivariable model. If included: independent predictor?
Ren, 2007, Heart and Soul study [85]	Stable CAD	639 (19%)	67	3	7% in those with mild diastolic dysfunction 11% in those with moderate-severe LVDD	Not investigated	No	Not performed	Yes, independence not reported
From, 2010, Olmsted County [82]	Diabetes mellitus	1760 (51%)	60	5	17% in those with diabetes 37% in those with diastolic dysfunction and diabetes	Not investigated	No	Not performed	Yes, not independent
Correa de Sa, 2010 [81]	Moderate or severe LVDD	82 (55%)	69	2	In those with moderate or severe LVDD 1.9% developed HF according to Framingham criteria and 31% developed signs or symptoms suggestive of HF (not explained by other conditions)	Peripheral vascular disease, hypertension	No	Not performed	No
Kane, 2011, Olmsted County [78]	General	1402 (51%)	61	6.3	7.8% in those mild diastolic dysfunction 12.2% in those with moderate-severe LVDD	Age, hypertension, diabetes, CAD, E/e′ ratio and LAVI	No	Not performed	No
Lam 2011, Framingham Heart Study [8]	General	1038 (61%)	76	11	23.8% of the population developed HF (~ 40% = HFpEF)	Renal impairment, airflow limitation and anemia	Yes	Not performed	Yes, independence not reported
Vogel, 2012, Olmsted County [79]	General	388 (57%)	67	3	11.6% in those with grade II to IV LVDD	Age, right ventricular systolic pressure, GFR < 60 mL/min per 1.73 m^2^	No	Not performed	No
Kuznetsova, 2014, FLEMENGHO [86]	General	793 (51.5%)	51	4.8	Incidence of cardiac event (including HF): 1.8% in normal LV diastolic function group 9.2% in impaired relaxation group 18.6% in elevated filling pressure	e′ tissue doppler velocity	No	Not performed	Yes, independence not reported
Yang, 2016 [80]	At risk for HF	428 (52%)	70	1.2	12.4% developed HF symptoms or died	Age, Charlson comorbidity score, GLS, LA enlargement	No	Not performed	Yes, not independent
Shah, 2017, ARIC [87]	General population (also including HF patients)	6118 (58%)	75.3	1.8	0.8% in group with stage A HF 3.4% in group with stage B HF	Structural abnormalities, systolic dysfunction, diastolic dysfunction	No	Not performed, sex and age specific echocardiography cut-offs were used	Yes, independence not reported
Pugliese, 2020 [83]	General population (also including HF patients)	304 (35%)	66	1.5	Incidence of HF hospitalization: 4.4% in group with stage A HF 15% in group with stage B HF	Resting NT- proBNP > 900 pg/mL, peak VO2 < 16 mL/kg/min, VE/VCO_2_ slope ≥ 36, peak PAPs ≥ 50 mmHg, and Δ B-lines > 10	No	Not performed	No, not independent
Bobenko, 2020, DIAST-CHF [88]	At risk for HF	851 (44%)	66	10	Signs or symptoms of HF: 54% in those without elevated filling pressures and 65% in those with elevated filling pressures	Not investigated	No	Not performed	Yes, independence not reported

Heart failure stages refer to the American College of Cardiology heart failure classification with stage A HF represents individuals at increased risk for HF without structural or functional heart abnormalities or heart failure signs or symptoms. Stage B represents individuals with structural abnormalities (such as abnormal diastolic function) in the absence of clinical signs and symptoms of HF. Stage C and D heart failure are marked by current or past evident heart failure signs and symptoms, accompanied by structural heart abnormalities

*Abbreviations*: *CAD*, coronary artery disease; *E/e′* ratio, the ratio of early mitral valve inflow (E) velocity divided by average e′; *GFR*, glomerular filtration rate; *GLS*, global longitudinal strain; *HF*, heart failure; *LA*, left atrium; *LAVI*, left atrial volume index; *LV*, left ventricle; *LVDD*, left ventricular diastolic dysfunction; *NT*-*proBNP*, N-terminal pro-brain natriuretic peptide; *peak PAPs*, peak systolic pulmonary artery pressure; *VE/VCO*_*2*_* slope*, minute ventilation/carbon dioxide production slope; *VO2 peak*, peak oxygen consumption

### Sex differences in risk factors for HF(pEF)

There is a significant knowledge gap on the exact mechanisms that are implicated in the progression from LVDD to HFpEF. We hereby review the risk factors associated with HFpEF, the knowledge on the mechanisms, and whether influences of sex are reported (see Fig. 1 and Table 3).

#### Age

Age is the strongest non-modifiable risk factor for LVDD and HF. In the Swedish Heart Failure Registry, women with HFpEF or HFrEF are approximately 4 years older compared to men with HFpEF or HFrEF [55]. Moreover, age is a stronger risk factor for HFpEF compared to HFrEF in a differential analysis from four observational studies, and this did not differ by sex [25]. Aging is an extremely complex process and has long been regarded as a topic beyond intervention. However, research into sex-specific aging mechanisms including sex-differences in telomere length, cellular senescence, and mitochondrial function preservation are all highly relevant when studying the progression from LVDD to HFpEF [100].

#### Hypertension

Hypertension is a major risk factor for HF with equal prevalence in both sexes [23]. Yet, the risk of HF in hypertensive women (HR = 3.35 (95% CI: 1.67, 6.73)) is more pronounced when compared to men (HR = 2.07 (95% CI: 1.34, 3.20)) [23]. Also, women with systolic blood pressure levels below the threshold of what has been considered the normal upper limit for decades (110–119 mmHg) seem to have an increased risk of HF (HR = 1.42 (95% CI: 1.11, 1.82)), which was not the case in men (HR = 1.02 (95% CI: 0.76, 1.38), *p*-value sex-interaction = 0.058) when using a SBP of 100–110 mmHg as a reference [24]. The importance of adequate hypertension treatment in HFpEF is not under debate, but sex-specific targets for blood pressure warrant further investigation as in women these may decrease all cardiovascular disease risk, not only HFpEF risk.

#### Diabetes

The prevalence of diabetes ranges from 4.3 to 28% in individuals with HF, and ~ 45% of the individuals with diabetes are women [22]. Diabetes increases the risk of HF more in women (HR = 3.73 (95% CI: 2.71, 5.15)) compared to men (HR = 1.82 (95% CI: 1.28, 2.30) [23]. In line with this, women with type 2 diabetes have higher HFpEF risk compared to men with type 2 diabetes [21]. This increased risk in women was recently also reported in a meta-analysis including 12 million individuals. Here, the discrepancy between risks was even larger for type 1 diabetes. The relative risk for HF was 5.15 (95% CI: 3.43, 7.74) for women and 3.47 (95% CI: 2.57, 4.69) for men with type 1 diabetes [22], but unfortunately, no distinction between HFpEF and HFrEF was made. These sex-differences in the association between HF risk and diabetes are possibly explained by worse microvascular function and lower coronary flow reserve in women with diabetes compared to men [101]. Furthermore, worse clinical outcomes found in HFpEF patients with insulin-treated diabetes versus diabetes not treated with insulin require further mechanistic investigation [102]. Possibly, changes in diabetes treatment regimens would benefit women most.

#### Obesity

Overweight is a global health problem and an acknowledged risk factor for HF. Sex differences in fat distribution exist, resulting in higher waist-to-hip ratios in men compared to women [103]. Women have a 4 to 29% higher prevalence of obesity compared to men, and there is high between-country variability in obesity prevalence [104]. The risk of HF and also specifically HFpEF is higher in obese women compared to obese men [19, 20]. In contrast, the association of BMI and other measures of adiposity (BMI, waist circumference, waist to hip ratio, body shape index, weight adjusted waist index, body roundness index, and relative fat index) with incident HFpEF and HFrEF or total HF is not different between women and men [103]. Overweight and physical inactivity go hand-in-hand, and exercise also protects obese individuals against cardiovascular disease [105]. We discuss the role of exercise in the section on treatment of HFpEF.

#### Smoking

The NHANES 1 study found that women who smoke have a 88% relative risk increase for HF compared to a 45% relative risk increase in men that smoke [89]. Smoking in this study was assessed between 1971 and 1975, and at that time the prevalence of current smoking was 40.7% in men and 31.1% in women [89], while 29% of men and 21% in women were active smokers in a more recent study that collected information on smoking status up to 2010 [19]. The latter did not confirm that daily smoking was a stronger risk factor for HF in women (HR women = 1.98 (95% CI: 1.77, 2.23), HR men = 1.93 (95% CI: 1.77, 2.10)) [19]. Hence, the evidence from a recent meta-analysis on coronary heart disease is convincing, showing that women who smoke have a 25% higher risk of coronary heart disease, while the mean consumption of cigarettes was not taken into account. Usually, cigarette consumption is higher in men than women, and taking this into account would have increased the risk in women even more [106]. A possible explanation for the observed increased risk of coronary heart disease is that women extract a greater quantity of toxic agents from cigarettes compared to men [107]. Also, women who smoke have lower levels of estrogens compared to women who do not smoke, and this may result in increased cardiovascular disease risk [108].

#### Ischemic heart disease

Ischemic heart disease is predominantly caused by epicardial coronary artery disease. Although intuitively the relationship of coronary artery disease and reduced ejection fraction is easily made, coronary artery disease is also a prevalent condition in HFpEF, especially in men. Presence of coronary artery disease, prior percutaneous intervention, and coronary artery bypass graft were all associated with hospital admissions for HFpEF in men only [90]. However, the presence of previous myocardial infarction is still more strongly associated to HFrEF than to HFpEF (HR HFrEF = 2.60 (95% CI: 2.08, 3.25) and HR HFpEF = 1.48 (95% CI: 1.12, 1.96)) [25].

Overall, hypertension, diabetes, and obesity are important HFpEF risk factors in women and are hypothesized to contribute to a state of systemic inflammation and endothelial dysfunction, leading to coronary microvascular rarefaction and stiffening of the heart [109, 110]. Additionally, given the higher prevalence of smoking and coronary artery disease in men compared to women, these are important risk factors to target to prevent the deterioration from LVDD to HFpEF in men. However, since smoking increases cardiovascular risk more in women, anti-smoking campaigns should also be tailored to women.

### Risk factors for HFpEF common in women

Apart from differences in the magnitude of the associations between risk factors and HFpEF in women and men, female-specific factors are often not studied but important to consider. We describe several female-specific and female-prevalent factors or disorders that might influence progression to HFpEF (see Fig. 1 and Table 3).

**Table 3:** General risk factors and risk factors for HFpEF common in women

**General risk factors for HFpEF**	
** Age**	Women with HFpEF are older than men with HFpEF [55], but age is not a stronger risk factor in women compared to men [25]
** Hypertension**	Women with hypertension have higher HF risk [23], HF risk increases at SBP ≥ 110 mmHg in women [24]
** Diabetes**	Two times stronger risk factor in women compared to men [22]
** Overweight**	Obesity is more prevalent in women and associated with higher HF risk in women compared to men [19, 20]
** Smoking**	Smoking increases CHD risk more in women [89], conflicting findings on HF [19]
** Ischemic heart disease**	Previous PCI and CABG are associated with HFpEF hospitalization in men [90]
**Risk factors for HFpEF common in women**	
** Auto-immune disease**	Established risk factor for CHD [91]. Research on HFpEF risk urgently needed [92]
** Pregnancy number**	Associated with diastolic- and exercise-RHC abnormalities [18, 93]. Research on HFpEF risk urgently needed
** Pregnancy complications**	PE increases HF risk [94], HPD is associated with concentric remodeling/LVH [95]
** Menopause**	Early menopause increases HF risk [96]. Higher estrogen levels at age 45 protect for HFrEF, but not for HFpEF [97]
** Mental health problems**	Antidepressant use is associated with CV-mortality [98]. Research on HFpEF risk urgently needed
** Migraine**	Predisposes to ischemic heart disease, stroke and AF, but not to HF [99]

*Abbreviations*: *CABG*, coronary artery bypass grafting; *CHD*, coronary heart disease; *CV*, cardiovascular; *HF*, heart failure; *HFpEF*, heart failure with preserved ejection fraction; *HFrEF*, heart failure with reduced ejection fraction; *LVH*, left ventricular hypertrophy; *PCI*, percutaneous coronary intervention; RHC, right heart catheterization

#### Auto-immune disease

There is a much higher prevalence of auto-immune disease in women compared to men (4:1 women to men ratio) that might contribute to systemic inflammation in HFpEF. This higher prevalence could be related to hormonal, genetic (e.g., escaping X-chromosome inactivation) and pregnancy factors [91, 111]. From an evolutionary perspective, women have a different immune system, tolerating pregnancy and placentation [111]. However, pregnancy on the other hand can also exacerbate auto-immune disease [112]. One conference abstract was published on a study attempting to quantify how much auto-immune diseases increase HF risk, stratifying for HF subtype and sex, but unfortunately detailed association measures were not provided [92]. Evidence on the cardiovascular consequences of auto-immune disease is sparse and mostly focusing on ischemic heart disease risk instead of HF [113]. As recommended by the ESC guidelines on cardiovascular disease prevention, auto-immune disease should be taken into account when considering initiation of preventive interventions [113].

#### Number of pregnancies

Women with four or more pregnancies have an increased risk of LVDD and decreased mitral annulus e′ velocity approximately 18 years after the latest delivery [18]. Potentially, reversible changes in each pregnancy may gradually lead to irreversible diastolic impairment. Also, in a cohort of HFpEF patients, women with ≥ 3 deliveries achieved a lower symptom-limited workload, developed a greater rise in pulmonary capillary wedge pressure indexed to workload, and had higher pulmonary vascular resistance than those with 0–2 births [93]. The authors hypothesized that pregnancies contribute to systemic inflammation, with possible mechanisms including adverse lipid profiles, upregulation of the renin–angiotensin–aldosterone system, and increased insulin resistance during pregnancy.

#### Pregnancy complications

The association of pregnancy complications such as hypertensive pregnancy disorders with atherosclerotic disease is well established [114]. A meta-analyses in almost 2 million women of which ~ 6% had pre-eclampsia found a four-fold increased risk of future HF (adjusted HR = 4.19 (95% CI: 2.09, 8.38)) [94], but this study did not distinguish between HFrEF and HFpEF. During pregnancy, circulating volume increases and a normal response to this is eccentric remodeling. However, women with hypertensive pregnancy disorders are susceptible to left ventricular concentric remodeling and hypertrophy, conditions that are sometimes persistent [115], and are common in HFpEF patients [116]. However, the mechanistic link between pregnancy complications and HFpEF still needs clarification.

#### Menopause and estrogen levels

The incidence of cardiovascular disease steeply increases in all women after menopause [117]. An early menopause increases the risk of ischemic heart disease risk [118], and of HF [96]. For each year that natural menopause is delayed, the annual risk of cardiovascular death decreases by 2% [119], and the annual risk of ischemic heart disease decreases by 3% [120]. One hypothesis is that this post-menopausal rise in cardiovascular disease incidence is attributable to a decline in estrogen levels. Estrogens are the primary female sex hormones and have been proposed to protect the heart from various forms of stress, including cytotoxic, ischemic, and hypertrophic stimuli [121]. In the 1990s, the landmark Women’s Health Initiative trial was conducted to investigate whether the protective effects of estrogens would be recovered when administering estradiol, or estradiol and progestin, to women without or with a history of hysterectomy, respectively. This research was terminated because women taking hormone replacement therapy showed an excess risk of venous thromboembolism and breast cancer and no protective effects on cardiovascular endpoints. However, small benefits were observed in “young” participants aged 50–59 years [122]. Afterwards, the timing hypothesis was brought up, which states that only peri-menopausal women benefit from estradiol replacement, as these women still have less severe atherosclerotic plaques compared to post-menopausal women in which estrogen administration would increase the risk of damage to the already vulnerable plaque. Some supporting evidence came from post hoc analyses of randomized controlled trials, but criticism was raised because of incomparable baseline characteristics [123]. Recently, the follow-up findings of women that were temporarily randomized to use post-menopausal hormone therapy or placebo were published [124]. There was no difference in the incidence of first HF hospitalization between the placebo and intervention arms, also not when stratifying for HFpEF and HFrEF [124]. In another, observational, study among women aged ≥ 45 years, a higher baseline estradiol level protected for HFrEF development (HR per SD increase in estradiol level = 0.60 (95% CI: 0.39, 0.93)), but not for HFpEF, during > 12-year follow-up [97]. Potentially, these protective effects are mediated through ischemic heart disease, which is still the main cause of HFrEF.

#### Mental health problems

The 2021 European Society of Cardiology guidelines for cardiovascular disease prevention recognize mental health problems and depression as important risk factors for cardiovascular disease. The use of antidepressants is associated with higher risk of all-cause mortality (RR = 1.27 95% CI: 1.21–1.34) and cardiovascular mortality (RR = 1.14, 95% CI: 1.08, 1.20) in HF patients [98]. However, few etiologic research has been conducted on this topic, and to our knowledge, no sex-specific data are available that study the association of mental health with HFpEF. Psychological stress and psychiatric disorders, however, are, among others, risk factors for Takotsubo syndrome [125]. This condition, characterised by transient left ventricular wall motion abnormalities beyond a single epicardial coronary artery distribution territory, while coronary arteries are not obstructed, is thought to result from sympathetically mediated microvascular dysfunction and women compose 90% of the cases. However, the female predominance in this syndrome and the role of estrogens in relation to younger age being a risk factor for a more complicated hospital admission is poorly understood [126].

#### Migraine

Migraine affects women approximately 3 times more than men and is more strongly associated with ischemic heart disease, stroke, and atrial fibrillation risk in women compared to men [99]. The risk of HF, however, is not significantly increased [99]. This is surprising since there are several common etiological links between HFpEF and migraine including endothelial dysfunction, a shared cardiovascular risk profile and comorbid inflammatory conditions [127]. Furthermore, increased stroke risk in migraine patients appears not to be mediated by atherosclerosis, since atherosclerosis is equally common in stroke patients with and without migraine [128]. Also in HFpEF patients, atherosclerotic lesions are less likely to explain ischemia since this is often a microvascular problem [129]. Future studies should explore whether female-prevalent disorders such as Takotsubo syndrome, HFpEF, and migraine have a shared vascular pathophysiology and whether potential therapeutic targets for these disorders are similar.

### Sex-differences in prognosis in women and men with HFpEF

Women and men with HF have equal mortality rates [72, 130], but the probability of re-hospitalization for HF is higher in women (34% re-admissions in women compared to 27% in men) [72]. Data on mortality and hospitalization, however, are not consistent. Three studies reported significantly better outcomes in women with HFpEF compared to men with HFpEF [55, 131, 132]. Also, women with HF were more frequently admitted for non-cardiovascular causes [130], and women hospitalized with HFpEF were at higher risk of poor post-discharge outcomes (adjusted HR = 1.54 (95% CI: 1.14, 2.07) than men [133], which may be due to high comorbidity burden in women. This high comorbidity burden together with a higher prevalence of obesity and worse diastolic and vascular function and greater exercise limitations might reflect different HFpEF etiologies and can partly explain the inconsistencies in prognostic studies [39, 131, 134, 135]. Additionally, women with HFpEF have a worse quality of life compared to men with HFpEF, and this is also consistently observed in the general community [134]. A lower quality of life in women is potentially attributed to a higher symptom burden, less social support, or more depression [134]. Additionally, women may perceive impairment as more severe compared to men [134]. Two community studies showed that a lower quality of life or lower self-rated health, respectively, are associated with asymptomatic LVDD [5, 136], and counter-intuitively, the age-adjusted association of self-rated health with LVDD was only significant in men (OR = 3.49 (95% CI: 1.0, 11.9)) [136].

### Sex-differences in HFpEF treatment response

After years of disappointing clinical trials, the first evidence-based HFpEF treatment has been found. Two trials on sodium-glucose co-transporter 2 (SGLT2) inhibition, studying empagliflozin and dapagliflozin, respectively, in HFpEF patients, were able to meet their primary endpoint of reducing cardiovascular mortality and HF hospitalization, in both sexes [65, 137]. At the moment, SGLT2 inhibition is recommended in the American HF guidelines (level of evidence 2A), and it is expected that European guidelines will follow soon [138]. Now that these pharmacological treatments for HFpEF become available, aggressive management of pre-clinical LVDD with the same drugs should be investigated, to prevent deterioration to HFpEF. Further current guideline recommendations include treatment with diuretics in congested HFpEF patients (level 1A of evidence) [38, 138], and the American guidelines also have a 2B level of evidence recommendation for treating selected HFpEF patients with sacubitril-valsartan, angiotensin receptor blockers, or mineralocorticoid receptor antagonists. Interestingly, although sacubitril-valsartan did not convincingly reduce the composite outcome of HF hospitalization and cardiovascular death in patients with HFpEF from the PARAGON-HF trial, sex appeared to modify the effect of treatment on the outcome. A benefit was indeed seen in women, in which the rate ratio for the primary outcome for sacubitril-valsartan versus valsartan was 0.73 (95% CI, 0.59–0.90), while in men, no benefit was reported (rate ratio = 1.03 (95% CI, 0.84–1.25)) [139]. Since the average ejection fraction is higher in women, it was hypothesized that a proportion of women in the trial had mild systolic dysfunction. This could represent a plausible explanation for the observed benefit of sacubitril-valsartan in women, considering that the drug has been clearly demonstrated to be effective in the presence of LV systolic dysfunction [139]. Another example of sex-specific treatment response to HF drugs comes from an exploratory post hoc analysis of the TOPCAT trial, showing a reduced risk of in all-cause mortality in women treated with spironolactone (HR = 0.66 (95% CI: 0.48, 0.90), while no effect was observed in men (HR = 1.06 (95% CI: 0.81, 1.39) [140]. A more pronounced protective effect on cardiac remodeling has been hypothesized as one of the contributing factors of the response to spironolactone in women. Sex-differences in pharmacokinetics and pharmacodynamics underpin these differences in treatment responses and have also been demonstrated for other HF drugs such as ACE-inhibitors, ARBs, and beta-blockers. Observational and routine health care data studies showed that women with HF are better off with lower doses of these drugs, bringing into question whether or not optimal medical treatment should rather be defined sex-specifically [141, 142]. Additionally, it should be noted that women were underrepresented in HFpEF trials testing drug therapies, and although post hoc analyses did not show effect modification by sex, those sub-analyses were underpowered and thus unlikely to detect sex differences.

#### Lifestyle interventions

Exercise training is recommended in all patients with chronic HF [38], and endurance training significantly improves health-related quality of life in HFpEF patients [143], while at the same time LVDD not significantly improves [143]. Worldwide, women are more often physically inactive compared to men, with high between-country variability [144]. Among 40,095 postmenopausal women without HF, those with the healthiest lifestyle (high levels of self-reported physical activity, eating a healthy diet, being non-smokers, and having a BMI between 18.5 and < 25.0 kg/m^2^) had the lowest HFpEF risk (adjusted HR = 0.23 (95% CI: 0.15, 0.35) compared to those with the worst lifestyle [145]. To our knowledge, sex-differences in the effect of lifestyle interventions in patients with or at risk for HFpEF have never been investigated. The positive effects of a healthy diet and exercise on HF hemodynamics have been suggested to be at least partly mediated by reduced inflammation and improved endothelial function [146, 147], as well as by improved heart rate reserve and improved muscle oxygen utilization [143]. Lifestyle interventions may represent an effective strategy to prevent or delay the progression of LVDD towards HFpEF in women at risk, as women are more prone to have an inactive lifestyle compared to men [144] (see Fig. 1).

### Pre-clinical research

Since there is a broad understanding that HFpEF is a multifactorial, multi-organ, multi-comorbidity syndrome, numerous pre-clinical models have been developed to understand disease mechanisms and to identify therapeutic targets. Over time, there has been a transition from simple single-hit models to multi-hit models involving age, a Western high fat/high sugar diet, diabetes, hypertension, hypercholesterolemia, and kidney dysfunction as stressors and/or comorbidities [148]. These models enable sex-specific and phenotype-specific research [148, 149]. However, a major drawback is the HFpEF definition. Many studies define disease outcomes based on structural and functional parameters, and the models actually represent extended LVDD models [149]. To overcome this, signs of congestion, such as lung weight, natriuretic peptide levels, and, ultimately, symptoms, should be taken into account. In our opinion, pre-clinical models are not fully suitable to study the natural progression of LVDD towards HFpEF, but especially aging and hypertension/kidney disease models provide opportunities to investigate the pre-clinical stage of HFpEF in a sex-specific way.

## Conclusion

Outstanding progress has recently been made when it comes to knowledge on LVDD and HFpEF as separate entities. However, there are still major gaps on mechanisms involved in the progression from LVDD to HFpEF which we hypothesize to be sex-specific. Established risk factors such as hypertension, diabetes, and obesity are more important in women. Potentially, we are overlooking female-specific and female-prevalent risk factors, and more research into pregnancy associated risk factors is needed. Women with HFpEF have a tendency to show poorer prognosis, including a lower quality of life, compared to men. Lifestyle interventions, including a more active lifestyle, could have larger benefits in reducing the risk of progression from LVDD towards HFpEF in women compared to men and require further investigation.

## Data Availability

Data sharing is not applicable to this article as no datasets were generated or analyzed during the current study.

## Abbreviations

ACE-inhibitor: Angiotensin-converting enzyme-inhibitor
BMI: Body mass index
CAD: Coronary artery disease
CVD: Cardiovascular disease
COPD: Chronic obstructive pulmonary disease
E/e′ ratio: The ratio of early mitral valve inflow (E) velocity divided by average e′
e′: Mitral valve annular early filling tissue Doppler velocity
GFR: Glomerular filtration rate
GLS: Global longitudinal strain
HF: Heart failure
HFpEF: Heart failure with preserved ejection fraction
HFrEF: Heart failure with reduced ejection fraction
HR: Hazard ratio
LA: Left atrium
LAVI: Left atrial volume index
LV: Left ventricle
LVDD: Left ventricular diastolic dysfunction
LVEF: Left ventricular ejection fraction
NT-proBNP: N-terminal pro-brain natriuretic peptide
OR: Odds ratio
PAPs: Peak systolic pulmonary artery pressure
RCT: Randomized controlled trial
SGLT-2-inhibitor: Sodium-glucose cotransporter-2- inhibitor
TR: Tricuspid regurgitation
VE/VCO_2_ slope: Minute ventilation/carbon dioxide production slope
VO2 peak: Peak oxygen consumption
